# Macroscopic T-Wave Alternans in QTc Prolongation: A Warning Sign Before Torsades de Pointes

**DOI:** 10.1016/j.jaccas.2026.108807

**Published:** 2026-07-29

**Authors:** Aishwarya Arunkumar Kamble, Ramy Doss, Jay Jaiswal, Ashwani Gupta

**Affiliations:** a Department of Internal Medicine, St Mary Medical Center, Langhorne, Pennsylvania, USA; b Department of Cardiology, Nazareth Hospital, Philadelphia, Pennsylvania, USA; c Department of Cardiac Electrophysiology, Cardiology, St Mary Medical Center, Langhorne, Pennsylvania, USA

**Keywords:** alcohol, electrocardiogram, ventricular fibrillation, ventricular tachycardia

## Abstract

**Case Summary:**

A 28-year-old man with alcohol use disorder presented with delirium tremens and electrocardiographic QT prolongation with macroscopic T-wave alternans, followed by torsades de pointes and ventricular fibrillation. Echocardiography demonstrated a dilated left ventricle with an ejection fraction <20%. The findings highlighted profound repolarization instability preceding malignant ventricular arrhythmias.

**Take-Home Message:**

Macroscopic T-wave alternans is an ominous electrocardiographic marker of profound repolarization instability and imminent polymorphic ventricular tachycardia, requiring immediate elimination of QT-prolonging factors, correction of electrolyte abnormalities, and prompt initiation of intravenous magnesium sulfate.

## Graphical Abstract

**Figure:**
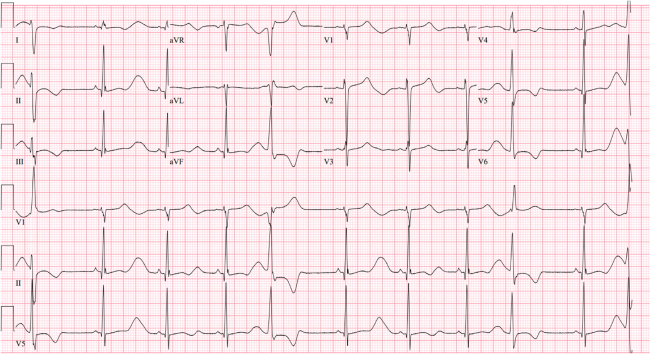

## Case Description

A 28-year-old man with alcohol use disorder was admitted with confusion. His vital signs were notable for sinus tachycardia and elevated blood pressure. He was disoriented to time and place, and demonstrated incoherent speech, consistent with alcohol withdrawal and possible delirium tremens. Laboratory evaluation showed severe lactic acidosis and ketoacidosis, hyperkalemia (potassium: 6 mEq/dL), elevated creatinine (1.5 m/dL), and elevated hepatic transaminases. Head computed tomography revealed an acute left frontoparietal subdural hematoma. Echocardiography demonstrated a severely dilated left ventricle with global hypokinesis and an ejection fraction of <20%. Telemetry revealed frequent premature ventricular complexes. Electrocardiogram (Figure 1) showed sinus rhythm, marked prolonged QTc, and unusual pattern of alternating T-wave morphologies with 3-beat periodicity. He also had frequent premature ventricular complexes in a pattern of quadrigeminy, with the QRS complex falling on the T wave (R-on-T phenomenon). Shortly after electrocardiogram acquisition, the patient developed torsades de pointes that degenerated into ventricular fibrillation, necessitating emergent defibrillation. He was subsequently started on intravenous magnesium sulfate.

**Figure 1:**
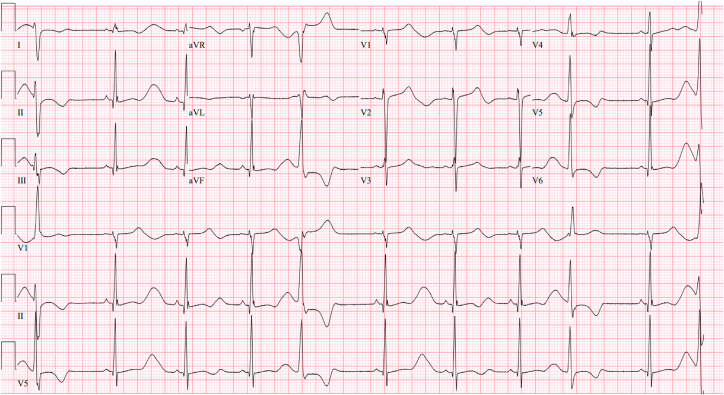
12-Lead ECG Demonstrating Normal Sinus Rhythm, Prolonged QT Interval, and Marked QT Dispersion and Premature Ventricular Complexes in a Pattern of Quadrigeminy with R-on-T Phenomenon There is beat-to-beat alternation with a 3-beat periodicity in T-wave amplitude and morphology, consistent with macroscopic T-wave alternans. ECG = electrocardiogram.

## Discussion

Torsades de pointes is a polymorphic ventricular tachycardia classically associated with QT interval prolongation, and it represents a life-threatening manifestation of ventricular electrical instability. The condition is typically multifactorial, arising from the convergence of predisposing substrate abnormalities and acute triggering factors. This case illustrates how alcohol withdrawal, severe metabolic derangements, acute neurologic injury, and underlying cardiomyopathy can synergistically precipitate malignant ventricular arrhythmias.

T-wave alternans (TWA) on electrocardiogram is identified by a repetitive pattern in T-wave amplitude or morphology, commonly with 2-beat periodicity and rarely with 3-beat periodicity, as demonstrated in this case. TWA reflects beat-to-beat variation in action potential duration and early after depolarizations that increase dispersion of repolarization, creating regional differences in refractoriness that facilitate reentry.^1^

Although QTc prolongation is present in many at-risk patients but rarely progresses to torsades de pointes, macroscopic TWA represents a rare but highly specific marker of imminent ventricular arrhythmia. QTc prolongation establishes the arrhythmogenic substrate through delayed repolarization, whereas TWA indicates critical dysfunction in intracellular calcium handling that actively triggers arrhythmia initiation.^2^

The recognition of TWA as a marker of arrhythmogenic risk has led to the development of microvolt TWA testing, a noninvasive tool used for risk stratification of sudden cardiac death in selected patient populations.^3^

## Funding Support and Author Disclosures

The authors have reported that they have no relationships relevant to the contents of this paper to disclose.

**Take-Home Message**

• Macroscopic T-wave alternans is an ominous electrocardiographic marker of profound repolarization instability and imminent polymorphic ventricular tachycardia, requiring immediate elimination of QT-prolonging factors, correction of electrolyte abnormalities, and prompt initiation of intravenous magnesium sulfate.
